# Pelvic venous and pulmonary artery extension in low-grade endometrial stromal sarcoma: a case report

**DOI:** 10.3389/fonc.2025.1641836

**Published:** 2025-09-23

**Authors:** Yi-Ling Li, Xin Sun, Ming-Zhu Ye, Chen Wang, Hui Cheng, Shan-Yu Huang, Xiao-Xiao Xi

**Affiliations:** Department of Obstetrics and Gynecology, The Third Xiangya Hospital, Central South University, Changsha, Hunan, China

**Keywords:** low-grade endometrial stromal sarcoma, vascular invasion, tumor thrombus, pulmonary embolism, uterine sarcoma

## Abstract

**Background:**

Vascular invasion is a hallmark of low-grade endometrial stromal sarcoma (LG-ESS), typically presenting a tumor thrombus within the parametrial vessels. However, extension of tumor thrombus into major vessels such as pulmonary artery is extremely rare. This case report presents an unusual manifestation of LG-ESS with intravascular tumor thrombi extending to the pelvic venous and pulmonary artery, highlighting the diagnostic challenges and clinical implications of vascular involvement.

**Case presentation:**

A 42-year-old female presented with prolonged menstruation. Imaging examination initially suggested multiple uterine fibroids, and hysteroscopic resection of a polypoid lesion revealed stromal hyperplasia, raising suspicion of an endometrial stromal nodule. One year later, the patient developed a pulmonary embolism and imaging showed disease progression. A hysteroscopic biopsy confirmed the diagnosis of LG-ESS. She subsequently underwent total abdominal hysterectomy (TAH) with bilateral salpingo-oophorectomy (BSO) and venous thrombectomy. Histopathological examination revealed LG-ESS with deep myometrial invasion and extensive intravascular tumor thrombus extending into the pelvic veins. The pathological stage was pIIA(FIGO). Postoperative chemotherapy with liposomal doxorubicin was administered, and follow-up pulmonary computed tomography angiography showed resolution of the emboli. No recurrence was noted at the six-month follow-up.

**Conclusions:**

This case highlights the diagnostic complexity of LG-ESS due to its nonspecific clinical presentation and imaging findings, especially in early stages. Rare vascular involvement, including pulmonary artery tumor embolism, poses a risk of misdiagnosis and highlights the importance of thorough histopathological evaluation. Early hysteroscopic intervention, accurate pathological assessment, and individualized adjuvant therapy are critical for optimizing outcomes in patients with LG-ESS exhibiting vascular extension. Informed consent for publication was obtained from the patient.

## 1 Introduction

Endometrial stromal sarcoma (ESS) is a rare malignant neoplasm arising from the endometrial stromal cells, accounting for approximately 0.2% of all uterine malignancies and 10% of all uterine sarcomas, with an estimated annual incidence of 1–2 cases per million women (1). According to the 2020 World Health Organization (WHO) classification, ESS is categorized into four subtypes based on the differentiation of tumor cells: endometrial stromal nodule (ESN), low-grade endometrial stromal sarcoma (LG-ESS), high-grade endometrial stromal sarcoma (HG-ESS), and undifferentiated uterine sarcoma (UUS) (2). Among these, LG-ESS has a comparatively favorable prognosis and is the second most common uterine sarcoma after leiomyosarcoma. However, precise epidemiological data remain limited. Tumor invasion is often observed in the parametrial vessels, but extension into major blood vessels such as the pulmonary artery is extremely rare (3).

In the present case, the patient was initially diagnosed clinically with leiomyoma. An initial hysteroscopic biopsy suggested possible LG-ESS. One year later, follow-up imaging revealed vascular invasion by the tumor, emphasizing the critical importance of adequate pathological sampling in the diagnosis of LG-ESS. Given the aggressive nature of the disease, early diagnosis and complete surgical resection are crucial.

## 2 Case presentation

**Figure 1:**
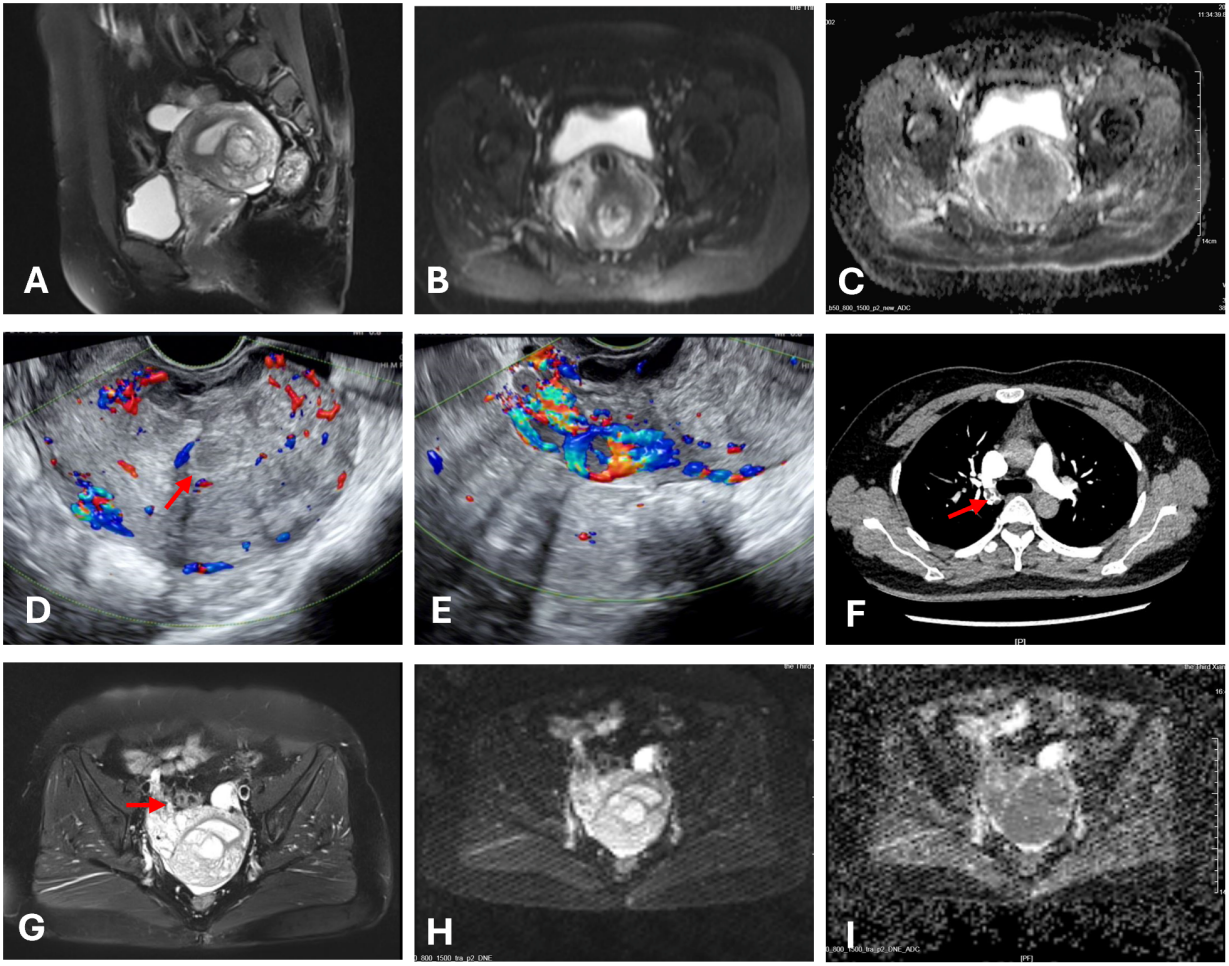
Images of the mass. The red arrow indicates the mass. **(A)** Multiple nodular lesions in the posterior uterine wall. **(B, C, H, I)** The tumor showed high signal on DWI and low signal on the ADC. **(D)** Ultrasonography shows multiple ill-defined hypoechoic nodules coalescing in the myometrium. **(E)** Diffuse linear hypoechoic nodules along arcuate vessels (anterior uterine wall) and right parametrial vessels, with increased blood flow signals. **(F)** CTA showed filling defects within the right pulmonary artery. **(G)** Nodular and tortuous tubular structures which shares similar MRI signals with the uterine mass were suggestive of angioleiomyoma.

**Figure 2:**
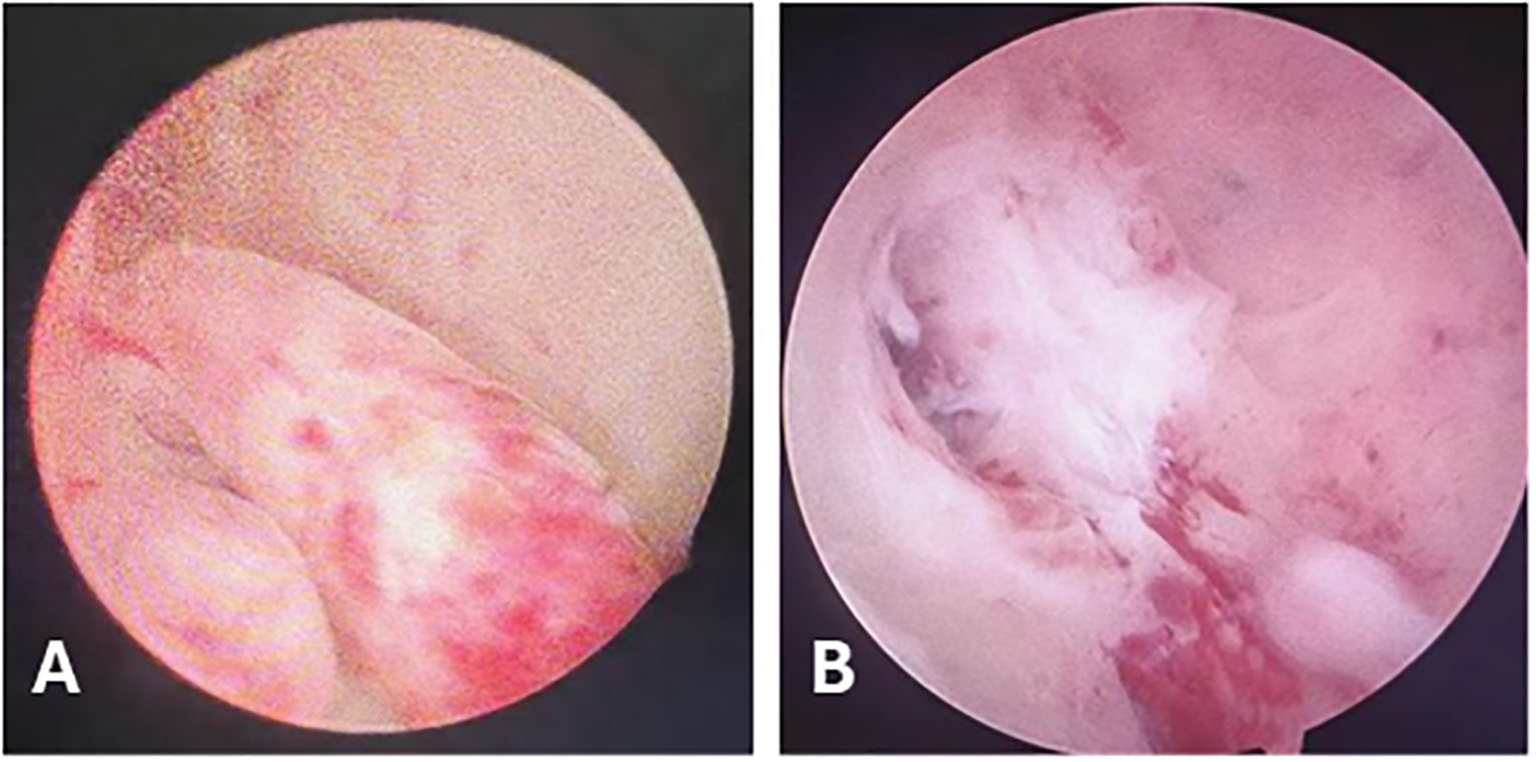
Hysteroscopic Findings. **(A)** Tongue-like polyps in the posterior uterine wall. **(B)** During resection, the polyp base was found to invade the myometrium.

In August 2023, a 42-year-old female patient was admitted to our hospital due to prolonged menstrual periods. Transvaginal ultrasound revealed a 30 × 30 mm hypoechoic nodule at the right wall of the uterine fundus, protruding approximately 20% into the uterine cavity, suggestive of fibroid. Magnetic resonance imaging (MRI) demonstrated multiple nodular lesions in the posterior uterine wall, the largest one measuring 28 × 26.2 × 26.4 mm and protruding into the uterine cavity (**Figures 1A–C**). These findings were consistent with multiple fibroids, including partially submucosal fibroids. Hysteroscopic examination revealed tongue-like polyps in the posterior uterine wall. The polyp was resected, however, the polypoid lesion exhibited poorly defined borders with the myometrium, indicating deeper myometrial involvement during resection (**Figures 2A, B**). Postoperative histological examination revealed nodular hyperplasia of stromal cells with focal proliferative endometrium within the mass. Endometrial stromal nodules or other neoplastic lesions could not be excluded. Postoperatively, the patient’s menstrual periods normalized, and did not adhere to the recommended further therapy and regular follow-up.

The patient was readmitted in September 2024 for an irregular follow-up. Ultrasonography revealed multiple hypoechoic nodules with ill-defined margins and a coalescent appearance within the uterine parenchyma. Diffusely distributed linear hypoechoic nodules were noted along the arcuate vessels of the anterior uterine wall and right parametrial vessels, with relatively abundant blood flow signals (**Figures 1D, E**). MRI revealed an increase in lesion size compared to previous imaging studies, with current dimensions of 58 × 46 × 52 mm. Nodular and tortuous tubular structures were observed in the right anterior uterine region and surrounding tissues, with mild T1 and prolonged T2 signals and marked enhancement on contrast imaging, which shares similar MRI signals with the uterine mass (**Figures 1G–I**). These imaging findings were suggestive of angioleiomyoma. The patient denied abdominal pain, distension, edema, or urinary symptoms. The patient had no significant prior medical history and reported no family history of malignancy. On physical examination, the uterus was enlarged to the size of a 2-month gestation, with firm consistency and no tenderness. No abnormalities were observed in bilateral adnexal regions. Laboratory findings showed a D-dimer level of 0.87 mg/L, fibrinogen level of 4.22 g/L, lactate dehydrogenase (LDH) of 166 U/L, and cancer antigen (CA)-125 of 9.88 U/mL. Routine blood and urine tests, electrocardiography (ECG), and echocardiography were all within normal limits.

Computed tomography (CT) pulmonary angiography (CTA), combined with abdominal CT, revealed multiple pulmonary emboli in the right pulmonary artery (**Figure 1F**), accompanied by filling defects in the right ovarian vein. Doppler ultrasonography of the bilateral iliac veins, lower extremity veins, and inferior vena cava revealed normal findings. Due to the patient’s fertility preservation request and the presence of an intracavitary protruding mass, hysteroscopic resection was initially performed. Intraoperative findings revealed multiple polypoid projections in the uterine cavity, with the largest lesion (approximately 1.5 cm in diameter) located on the posterior wall. A myoma-like protrusion with approximately 20% intracavitary extension was also identified on the posterior wall. Postoperative histological examination revealed nodular hyperplasia of the endometrial stroma with focal infiltration into the myometrium, while significant cytological atypia was absent. Immunohistochemistry showed positive expression of CD10, estrogen receptor (ER, 90%), and progesterone receptor (PR, 95%), weakly positive Cyclin D1 in approximately 20% of tumor cells, negative staining for desmin, smooth muscle actin (SMA), h-caldesmon, and cytokeratin. Ki67 index (15% in hotspot areas). These findings raised strong suspicion for LGESS. A multidisciplinary team (MDT) discussion was convened following the second hysteroscopic procedure. The uterine mass was considered highly suggestive of LGESS. Regarding the pulmonary embolism, which presented on CTA as multiple, irregular, filling defects with heterogeneous enhancement on post-contrast, these features are more consistent with tumor thrombus rather than bland thrombus. However, considering the absence of respiratory symptoms and the limited extent of involvement, surgical embolectomy was not pursued.

**Figure 3:**
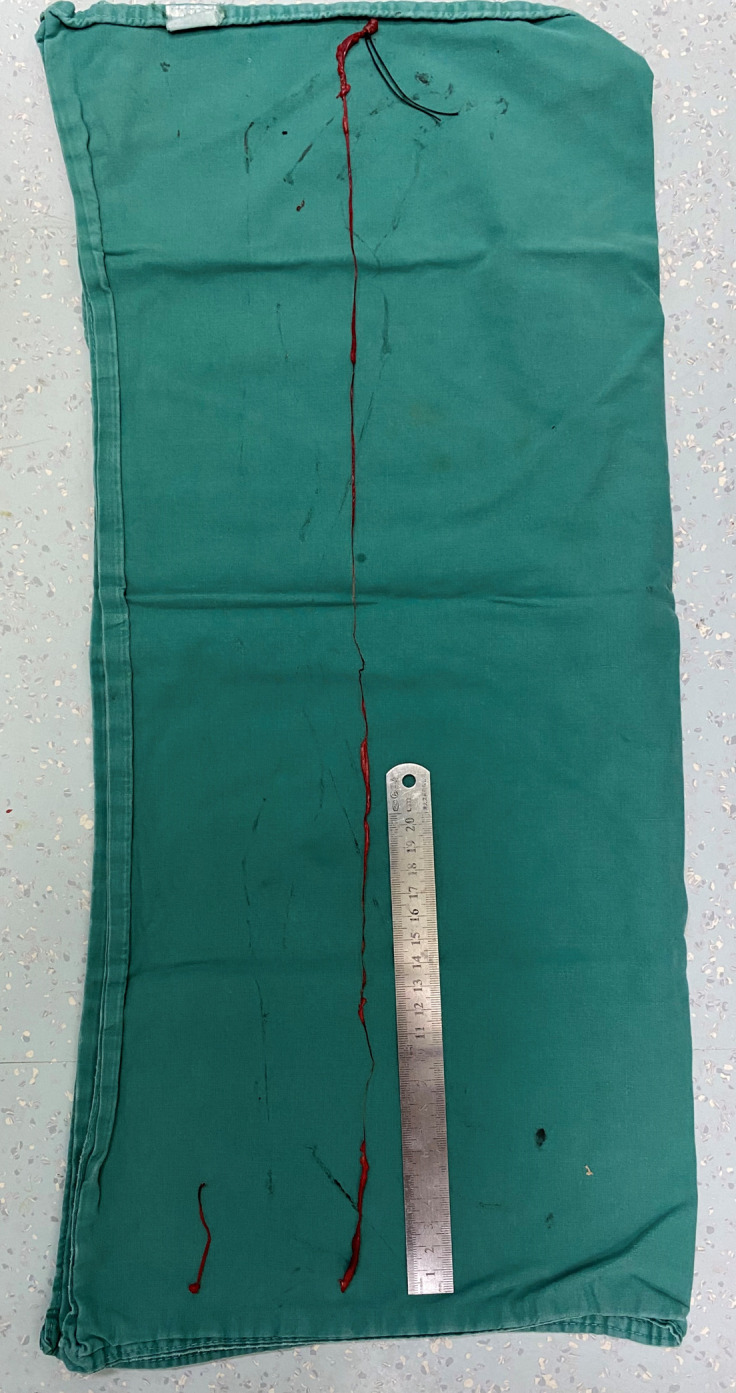
Two segments of tumor thrombus were retrieved, the shorter segment (approximately 5.5 cm in length) was derived from the ovarian vein, the longer one showed irregular caliber (1–8 mm in diameter) with a smooth, intact distal end, confirming complete retrieval.

On September 26, 2024, the patient underwent total abdominal hysterectomy with bilateral salpingo-oophorectomy and venous thrombectomy. The preoperative diagnoses were: 1. Endometrial stromal sarcoma? 2. Endometrial polyps 3. Scarred uterus 4. Gonadal vein thrombus? 5. Pulmonary embolism. Intraoperative findings revealed a uterus enlarged to the size of a 2-month gestation, with a firm, globular appearance. Both ovaries and fallopian tubes appeared grossly normal. The suspensory ligament of the right ovary was notably thickened, and a cord-like structure extending superiorly was palpable. Dissection of the ovarian vein up to the renal vein level was performed by vascular surgeon, followed by clamping and ligation inferior to the renal vein. An irregular, thickened, flesh-colored embolus measuring approximately 65 cm in length with a smooth distal end was meticulously extracted (**Figure 3**). (The tumor thrombus could be stretched significantly beyond the anatomical length of the involved vein due to its soft consistency.) The patient received perioperative anticoagulation therapy with enoxaparin at a dose of 4000 IU subcutaneously every 12 hours.

**Figure 4:**
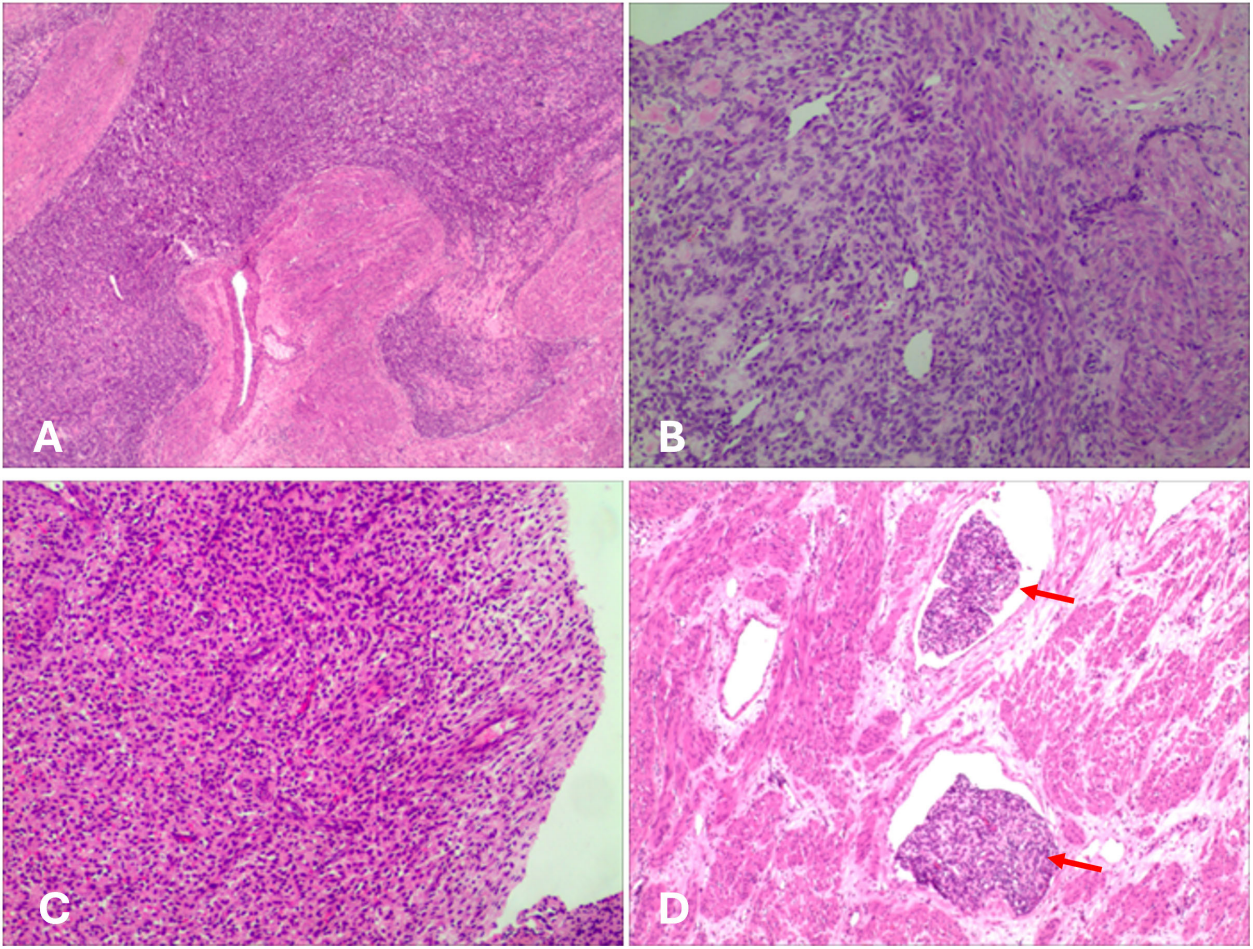
Histological image. **(A)** The tumor cells infiltrate the myometrium. **(B, C)** The tumor cells exhibit round to oval morphology, with scant cytoplasm and hyperchromatic nuclei, resembling proliferative-phase endometrial stroma. Focal hemorrhage and necrosis were observed. **(D)** Numerous tumor thrombi (red arrows) were observed within vessels.

**Figure 5:**
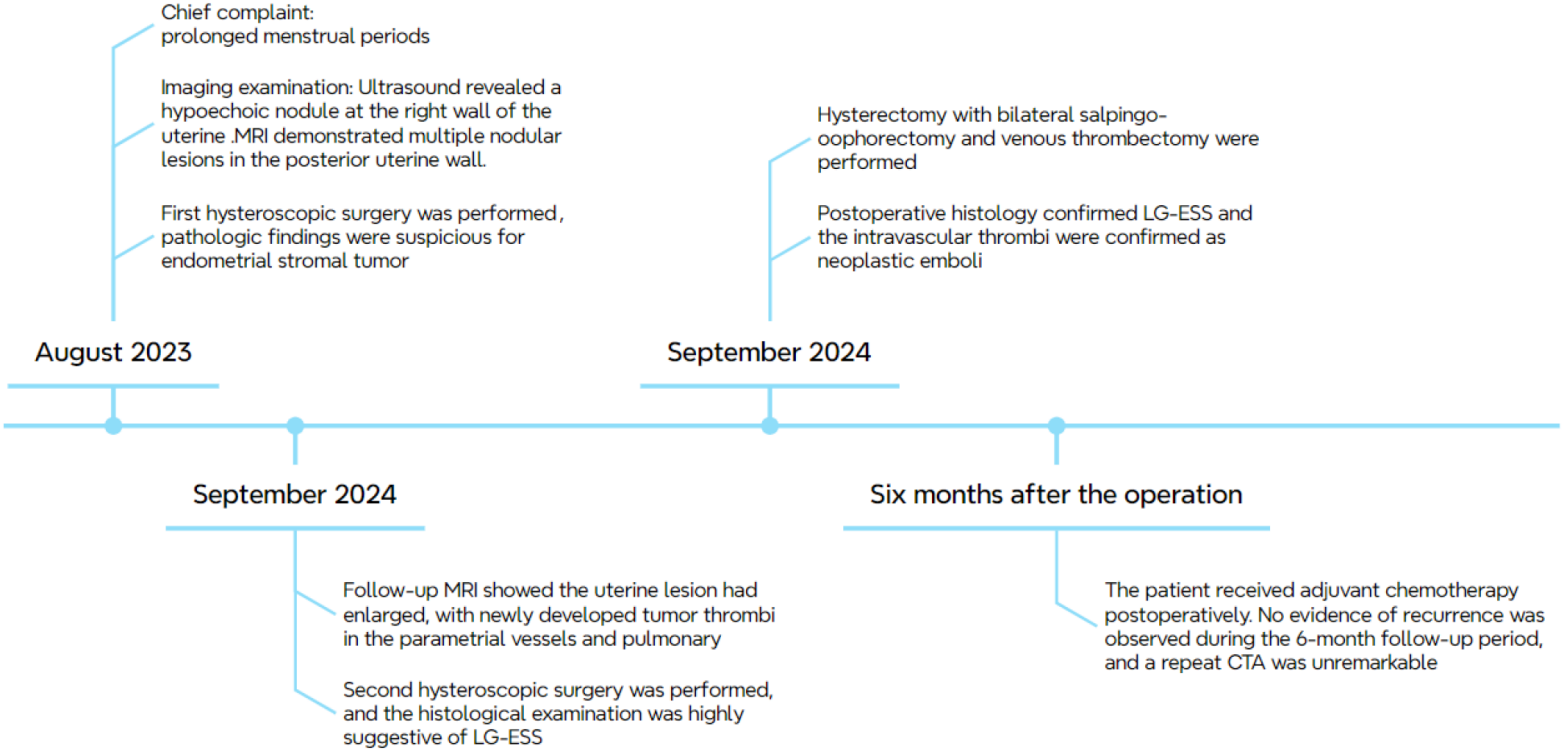
The clinical management pathway of the patient.

Gross pathological examination of the hysterectomy and bilateral adnexectomy specimens revealed a uterus with grayish-white, soft tissue consistency and multiple nodules within the myometrium, the largest measuring approximately 5 cm in diameter. Postoperative histology confirmed LG-ESS with focal hemorrhagic necrosis. The tumor demonstrated deep myometrial invasion (>50% of myometrial thickness) and extensive intravascular tumor thrombi. Metastatic tumor involvement was observed in the right ovary, along with tumor thrombi in the parametrial vasculature. The intravascular thrombi were confirmed as neoplastic emboli with associated degenerative necrosis (**Figure 4**). The pathological stage was determined as pIIA (34). Immunohistochemical analysis revealed strong positive expression of CD10, Wilms tumor 1 (WT1), ER, and PR, minimal focal expression of SMA, desmin, and Cyclin D1,5%+ Ki-67 labeling index. Negative staining for of h-caldesmon and cytokeratin. The results of IHC are summarized in **Table 1**. Given the aggressive invasion of tumor, the patient received postoperative chemotherapy (liposomal doxorubicin, 40mg/m^2^). Follow-up pulmonary CTA showed normal findings, and no recurrence was observed after six months. The clinical management pathway for this patient is illustrated in **Figure 5**.

**Table 1:** Summary of immunohistochemical (IHC) results.

Marker	Intensity/ percent positive
CD10	(+++)
Wilms tumor 1 (WT1)	(+++)
ER	(+++)
PR	(+++)
SMA, desmin, Cyclin D1	(minimal focal +)
h-caldesmon and cytokeratin	(-)
Ki-67	5%

## 3 Discussion

The diagnosis of LG-ESS is challenging due to its nonspecific clinical presentation and inconclusive imaging findings in the early stages. Ultrasonography and MRI often fail to differentiate LG-ESS from benign conditions such as leiomyoma, and the reported preoperative biopsy misdiagnosis rate is as high as 40% (4). In this case, the MRI revealed multiple nodules in the posterior uterine wall that were isointense on T1WI, slightly hyperintense on T2WI, showing high signal on DWI and low signal on ADC. These findings are helpful in differentiating the lesions from leiomyoma. A tentative pathological diagnosis was made during the first hysteroscopic procedure, based on the observation of deep myometrial invasion. This emphasizes the importance of obtaining sufficient tissue during sampling for accurate diagnosis. Although vascular invasion is a recognized feature of LG-ESS, involvement of major vessels such as the pulmonary artery, as observed in this case, is exceedingly rare. In this case, the tumor cells of LGESS appear round to oval, resembling those of proliferative-phase endometrial stromal cells and are characterized by infiltrative growth into the myometrium, this contrasts with the fascicular structure of bland, spindle-shaped smooth muscle cells characteristic of IVL. Moreover, the IHC strong positivity for CD10, ER, and PR, and negativity for h-caldesmon, which represents a key characteristic of LGESS. The focal and weak positivity for SMA and desmin may indicate smooth muscle differentiation in the tumor.

The diagnostic difficulty of LG-ESS arises from several factors. First, its clinical manifestations, including abnormal uterine bleeding, pelvic pain, or mass effect, are nonspecific. Second, imaging has limitations: although MRI is the preferred imaging modality and may reveal characteristic T2 hyperintensity with “worm-like” nodules, differentiating these features from benign lesions remains difficult (5). Third, histological and immunohistochemical evaluation can be hindered small or fragmented biopsies, which limit the assessment of deep myometrial invasion. The morphological overlap between LG-ESS and endometrial stromal nodules (ESN) complicates diagnosis, especially when tumor margins are poorly defined. Both LG-ESS and ESN typically express CD10, ER/PR, and interferon-induced transmembrance protein 1 (IFITM1) and are negative for Cyclin D1, desmin, SMA, and h-caldesmon, which can increase diagnostic uncertainty (6). Lastly, clinical recognition of LG-ESS is often delayed, due to its rarity and indolent progression, LG-ESS is frequently omitted from the differential diagnosis, and patients may not follow up once symptoms improve.

Vascular invasion is a key feature of LG-ESS. Worm-like tumor thrombi are typically found in the parametrial veins, however, extension into major vessels is extremely rare. Owing to its atypical clinical presentation, such cases may be misdiagnosed as intravenous leiomyomatosis (IVL). The incidence and prognosis of LG-ESS involving major blood vessels remain unclear due to the rarity of such cases. When tumor thrombi extend into the inferior vena cava or heart, patients may present with abdominal pain, back discomfort, lower limb edema, or dyspnea (1). There are approximately 46 cases of LG-ESS with major vessel or cardiac extension have been reported in the English literature (1, 7–26). Renzulli et al. reviewed 19 patients with advanced LG-ESS involving tumor thrombi in major blood vessels (9). A total of 18 patients underwent surgical treatment, with most requiring tumor thrombectomy via vena cava incision and right atriotomy. In one case, tumor thrombus in the inferior vena cava disappeared completely following neoadjuvant chemotherapy, obviating the need for surgical intervention. Among 10 patients who underwent radical tumor resection, nine remained recurrence-free during a mean follow-up of 2 ± 1.3 years. It is currently believed that tumor thrombi form via invasion of the ovarian vein and subsequent migration into the inferior vena cava. In the Renzulli review, at least six cases appeared to have originated from the ovarian veins. Similarly, in the present case, tumor thrombi were observed within the ovarian veins.

Total abdominal hysterectomy (TAH) with bilateral salpingo-oophorectomy (BSO) remains the primary treatment for early-stage LG-ESS. In cases with vascular involvement, complete thrombectomy is essential to relieve symptoms and prevent tumor recurrence (27). Single stage procedure (often involving a collaborative team of gynecologic and cardiovascular surgeons) is favored when feasible to achieve complete resection in one operation, while a two-stage approach may be safer for patients with widespread extension or limited tolerance for single surgery. Cardiopulmonary bypass (CPB), with or without hypothermic circulatory arrest, has been effectively employed in resections involving the inferior vena cava or heart (28). With the aid of cardiopulmonary bypass, the surgical team was able to remove the intravascular mass under direct vision and safely repair the vein (7). In our case, tumor thrombi extending between the ovarian and renal veins were completely removed to achieve maximal tumor clearance.

Although LG-ESS generally has a favorable prognosis, approximately 37%–60% of patients experience late-stage tumor recurrences (29). For patients with stage I disease, surveillance may be sufficient postoperatively. In contrast, hormonal therapy is the preferred adjuvant treatment for stage II–IV cases. Agents such as aromatase inhibitors, progestogens, and gonadotropin-releasing hormone (GnRH) analogs may reduce recurrence risk, although they have not shown significant impact on overall survival (OS) (30). The role of radiotherapy remains controversial. For patients with advanced-stage disease in whom hormonal therapy is ineffective or contraindicated, external beam radiation therapy (EBRT) and vaginal brachytherapy (BT) may be considered, balancing potential benefits with individual tolerability (31).

Few studies have investigated the role of chemotherapy in LG-ESS. Aunivariate analysis by Feng et al. indicated that chemotherapy may prolong the progression-free survival in early-stage LG-ESS cases (32). However, a retrospective cohort study by Zhou et al. found no significant difference in the 10-year recurrence rate between patients who received chemotherapy postoperatively (22.6%) and those who did not. Lymph node resection, ovarian preservation, and adjuvant therapies (chemotherapy, radiotherapy, or hormonal treatment) have shown no significant impact on disease-free survival (DFS) in LG-ESS (33). Thus, chemotherapy is not generally considered a first-line adjuvant treatment for LG-ESS. In the present case, given the patient’s imaging findings and clinical history, the pulmonary artery embolus was suspected to be a tumor thrombus. Therefore, the indication for adjuvant chemotherapy was expanded, and individualized treatment was provided to reduce tumor burden. Chemotherapy regimens included gemcitabine/docetaxel, doxorubicin/ifosfamide, and doxorubicin/dacarbazine. However, there is no optimal chemotherapy regimen. Despite initial response is encouraging, longer follow-up is necessary to assess durable disease control includes regular assessment of symptoms, physical examinations, transvaginal ultrasound (TVUS), and annual CT scans of the chest, abdomen, and pelvis. Insufficient follow-up time was also a limitation in this case.

## 4 Conclusion

This case highlights the diagnostic challenges of LG-ESS due to its nonspecific symptoms and imaging findings, as well as the importance of adequate histopathological sampling for timely and accurate diagnosis. The novelty of this report lies in the rare presentation of LG-ESS with tumor thrombus extending into the pulmonary artery, a manifestation that is exceedingly uncommon and easily misinterpreted as thromboembolism of non-neoplastic origin. Such vascular involvement may lead to diagnostic confusion and delayed management. Complete surgical resection remains the cornerstone of treatment, and multidisciplinary planning is essential, particularly in cases with extensive vascular extension. Long-term follow-up is critical due to the potential for late recurrence, and adjuvant therapy should be tailored based on disease stage, extent of involvement, and individual patient factors.

## Data Availability

The original contributions presented in the study are included in the article/supplementary material. Further inquiries can be directed to the corresponding author.
